# Dielectrophoresis Structurization of PZT/PDMS Micro-Composite for Elastronic Function: Towards Dielectric and Piezoelectric Enhancement

**DOI:** 10.3390/ma14154071

**Published:** 2021-07-21

**Authors:** Giulia D’Ambrogio, Omar Zahhaf, Minh-Quyen Le, Jean-Fabien Capsal, Pierre-Jean Cottinet

**Affiliations:** Univ. Lyon, INSA-Lyon, LGEF, EA682, Electrical Department, Ladoua Campus, F-69621 Villeurbanne, France; giulia.dambrogio@insa-lyon.fr (G.D.); omar.zahhaf@insa-lyon.fr (O.Z.); minh-quyen.le@insa-lyon.fr (M.-Q.L.); jean-fabien.capsal@insa-lyon.fr (J.-F.C.)

**Keywords:** ferroelectric composites, piezoelectric sensor, health monitoring, 1–3 connectivity, dielectrophoresis, smart materials

## Abstract

Piezoelectric materials have been used for decades in the field of sensors as transducers and energy harvesters. Among these, piezoelectric composites are emerging being extremely advantageous in terms of production, costs, and versatility. However, the piezoelectric performances of a composite with randomly dispersed filler are not comparable with bulk ferroelectric ceramics and electroactive polymers. In order to achieve highly performing and low-cost materials, this work aims to develop flexible composites made of Lead zirconate titanate (PZT) filler in Polydimethylsiloxane (PDMS) matrix, with a specific internal structure called quasi-1–3 connectivity. Such a structure, comprising particles arranged in columns along a preferred direction, is performed through dielectrophoresis by applying an alternating electric field on the composite before and during the polymerization. The developed flexible material could be introduced into complex structures in various application fields, as sensors for structural monitoring.

## 1. Introduction

Piezoelectric composites made of ferroelectric ceramic fillers embedded in polymeric matrix are commonly used as transducers, actuators, and sensors. Thanks to the combination of the constituent phases, piezocomposites exhibit good mechanical properties and piezoelectric activity [1,2,3,4,5,6]. By properly combining particles and polymer matrix, it is possible to tailor the features of the final material and thus to develop sensors for specific application [7,8,9]. By choosing a flexible matrix, piezoelectric composites can be less fragile than bulk ceramics, such as bulk lead zirconate titanate (PZT) and barium titanate (BaTiO_3_) [10]. Moreover, the appropriate selection of a ferroelectric filler results in materials operating at high temperatures, and therefore more advantageous than classical electroactive polymers like polyvinylidene difluoride (PVDF), P(VDF-TrFE) copolymer, and P(VDF-TrFE-CTFE) terpolymer [11,12,13,14,15,16,17]. Additional advantages are the low cost, ease of manufacture and reduced polarization electric field compared to common electroactive fluoropolymers [18]. In view of all these aspects, piezoelectric composites are thriving as transducer in different fields such as aerospace, automotive, nuclear, and medical. In the industrial sector they play a fundamental role in the structural health monitoring. This process consists in the examination of the safety and durability of structures, and in the detection of any damage or defect that could lead to catastrophic failures. Among all the non-destructive tests, the use of sensors based on piezoelectric composites is an interesting solution. Their main advantage would be that, placed permanently on a structure, they could allow an online monitoring during the service life [19,20,21]. Piezoelectric composite sensors are also widely used in the medical fields for high frequency ultrasound imaging, in infusion, insulin pumps, respiratory monitoring, in blood pressure monitoring equipment and surgical fluid management systems [22,23,24,25,26,27,28].

The arrangement of the phases, named as connectivity, determines the performance of these composites. Different possible arrangements of the phases can be exploited to tailor the piezoelectric properties of the composites [25,29]. It is thus possible to design a proper structure within the material to enhance the efficiency of the sensor [1,30,31]. Several studies demonstrate that a columnar arrangement of the fillers within the matrix, named 1–3 connectivity, improves the piezoelectric response along the alignment direction, with respect to a composite with filler randomly dispersed, named 0–3 connectivity [32,33,34,35]. 1–3 connectivity can be designed through several techniques such as rod placement, dice-fill, ultrasonic cutting, laser machining, co-extrusion, tape lamination, and fiber insertion method [3]. Among the various existing methods, electric field structuration via dielectrophoresis emerges as a promising process: an electric field is applied to the sample before (at ambient temperature) and during the curing process (at high temperatures) in order to drive the particles into a columnar structure [25,34,36,37,38]. The high temperature applied in the second stage of the process fixes the build-up design. Usually, the electric field distribution in a composite is non-homogeneous due to the discrepancy in permittivity between the matrix and the particles. This leads to a generation of dipoles in the ceramic particles, which, as a consequence, attract and repulse each other’s by electrostatic force, depending on their reciprocal position. When the matrix is cured under the electric field, the columns maintain their position to form a chain-like structure. This method is particularly convenient because of its easy processability, i.e., the possibility to be integrated in additive manufacturing based 3D printing technology [21,39,40]. In 1–3 connectivity, the particles redistribute themselves along columns, creating anisotropy in the material. The particles within the chain are tightly packed and experience strong interactions in the aligned direction [41]. A further benefit of the structuration is a reduction of the poling electric field required to trigger the piezoelectric activity. The distribution of the ceramic phase in parallel columns provokes a more effective distribution of the electric field within the composite, thus a better piezoelectric coupling. During the poling, the matrix shields less the electric field in an aligned 1–3 matrix, as opposed to a random 0–3 counterpart, where the shielding does not allow functional poling unless high electric fields are applied [42].

In this work, we demonstrate strong benefit of material structuration via dielectrophoretic technique. A comparison of both 0–3 and 1–3 connectivity is performed via Scanning electron microscopy (SEM) analysis, together with dielectric and piezoelectric characterizations. Experimental data are correlated to analytical models. In addition, thermal stability and high temperature X-ray diffraction analyses are investigated, confirming good piezoelectric response even at 200 °C. Dielectrophoresis reveals to be frequency dependent, which might affect the alignment’s degree of the piezoelectric phase. This analysis allowed to select the best parameters for achieving 1–3 PZT/PDMS composite.

## 2. Materials and Methods

### 2.1. Sample Preparation

**Figure 1:**
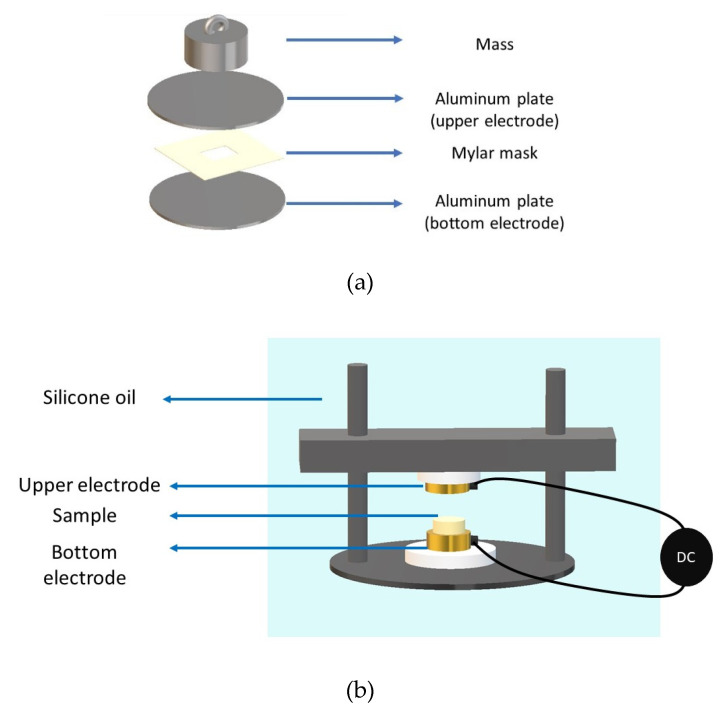
(**a**) Set up developed to produce the composites. (**b**) Poling set up.

Lead zirconate titanate is an inorganic compound with the following chemical formula Pb[Zr_x_Ti_1−x_]O_3_ (0 ≤ x ≤ 1). In literature it is usually simplified as PZT, which includes all the possible types of this compound. In this research, a commercial PZT 52/48 was used, with an average size of 3.8 µm, and a mass density (ρ) of 7600 g cm^−3^. The polymer used was linear vinyl terminated poly(dimethylsiloxane) Sylgard 184 elastomer, purchased at Dow Corning^®^. It was crosslinked with the curing agent by a ratio of 10 (base): 1 (curing agent). Powder and Sylgard base were weighted and mixed in a ultrasonication mixer for 10 min under I Type Cabinet B -ADS air Clean. The cross-linker was added in the mentioned ratio and mechanically mixed for 1 min. The mixture was dried under vacuum at 23 °C for 30 min in Memmert Vacuum oven. The blend was then poured into a mold consisting of a mylar spacer 250 µm-thick and a 3 × 3 cm^2^ surface of cut-out positioned between an upper aluminum plate (placed under a 500 g mass), and a bottom aluminum plate. Aluminum plates played a role of electrodes, from which a structuring field created 1−3 connectivity (Figure 1a). The mold was placed in an oven. For 0–3 composite, the oven was immediately switched on at 150 °C during 1 h. For 1–3 composites, the aluminum plates were connected to a voltage amplifier coupled with a sinusoidal wave generator. An AC electric field of 2 V µm^–1^ amplitude and 2 kHz frequency was applied for 15 min at room temperature. Later, the oven was started at 150 °C and the blend was left cooking under external electric field for 1h. The volume fraction prepared were 3.2% vol, 12% vol and 24% vol. Before performing the polarization, all the samples were sputtered with gold, to produce circular electrodes of 0.8 mm diameter and 25 nm thick on the top and bottom surfaces. The samples were then submerged in a silicone oil bath at 100 °C and poled for 30 min under a DC electric field of 20 V µm^−1^, which was found to be close to the coercive field of 1–3 composite at 24% vol (Figure 1b). The choice of a DC poling procedure instead of a bipolar polarization is related to the properties of the composite, which usually consists of two components: polymer matrix and ceramic filler. Considering the typical hysteresis curve of Current vs. Electric field of an alternating bipolar poling, the capacitive behavior of the polymer would mask the ferroelectric behavior of the filler. As a matter of fact, the ferroelectric current is quite small with respect to the capacitive component of the polymer [18,43,44,45].

### 2.2. Scanning Electron Microscopy

Scanning electron microscopy (SEM) was performed via *Hitachi FlexSEM 1000II* model (Hitachi High-Tech Corporation, Tokyo, Japan), with the aim of analyzing the morphology of PZT particles and visualizing the phases configuration in 0–3 and 1–3 connectivity at 1% vol. Image-J software (Version 1.44, National Institutes of Health, Bethesda, MD, USA) was then employed to determine the size distribution and the real shape of the particles.

### 2.3. Dielectric Spectroscopy

Dielectric test was performed through *Solarton spectrometer 1296A Dielectric Interface System* (1255, Oak Ridge, TN, USA) to obtain the dielectric permittivity of PZT/PDMS 0–3 and 1–3 composites. The real part of the dielectric permittivity at 1 kHz was recorded as a function of the volume content.

### 2.4. Piezoelectric Sensitivity

Piezoelectric charge coefficient, d_33_ (pC/N), was measured with APC d_33_ m. The piezoelectric sensitivity was measured relied on the Berlincourt method: a sample was clamped between two probes and subjected to a force at 110 Hz and a variation of 0.25 N. Through the upper probe, a static preload force was applied.

### 2.5. Thermal Analysis and X-ray Diffraction

With the aim of understanding the piezoelectric activity of the composites at high temperature, a thermal analysis was performed. The samples were annealed at different temperatures (50 °C, 100 °C, 150 °C, and 200 °C) for 10 min after poling (following the procedure reported previously in Section 2.1). Subsequently, after each annealing step, the piezoelectric charge coefficient, d_33_, was measured. This analysis was coupled with an X-ray diffraction study, performed at different temperatures (25 °C, 150 °C, and 200 °C), in order to understand the relationship between the piezoelectric activity and the crystalline phases in the ceramic filler. The XRD study was carried out using Philips X’Pert MPD diffractometer (Philips, Amsterdam, Netherlands) with carbon filtered CuKa (1.5406 Å) source.

### 2.6. In-Situ Microscopy

**Figure 2:**
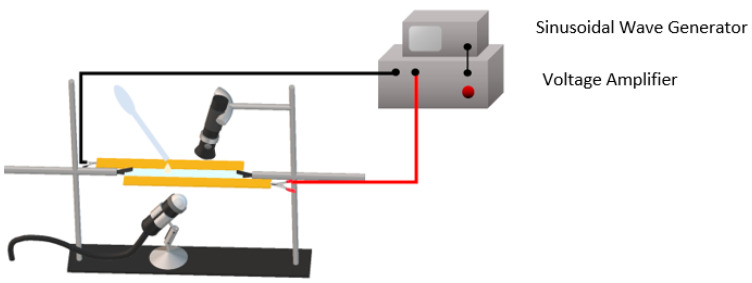
In-situ microscopy set up.

In situ microscopy was the final step required to investigate the process of structuring. Among all the parameters that affect dielectrophoretic technique, this work focused on the frequency dependency. A blend of particles and polymer with a volume content of 1% vol was prepared, without launching the polymerization stages. A portion of the blend was collected with a dropper and placed on a microscopy glass, between two copper electrodes previously installed. The electrodes were connected to a voltage amplifier coupled with a wave generator. A flashlight was positioned above the glass, while a USB-microscope was placed below it and connected to a computer. The observations lasted 15 min at a fixed electric field amplitude of 2 V µm^−1^, while different frequencies of 2 kHz and 2 Hz were applied. The experimental set up is illustrated in Figure 2. Frames at 0, 5, 10, and 15 min were taken from the videos for the two different observations, and the pictures were qualitatively compared.

## 3. Results and Discussion

### 3.1. SEM Observation

**Figure 3:**
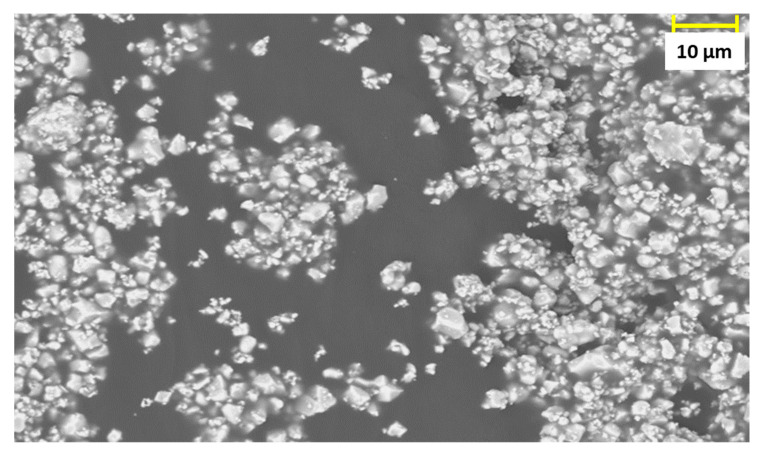
SEM picture, taken in back scattered electrons mode (BSE) of PZT fillers.

**Figure 4:**
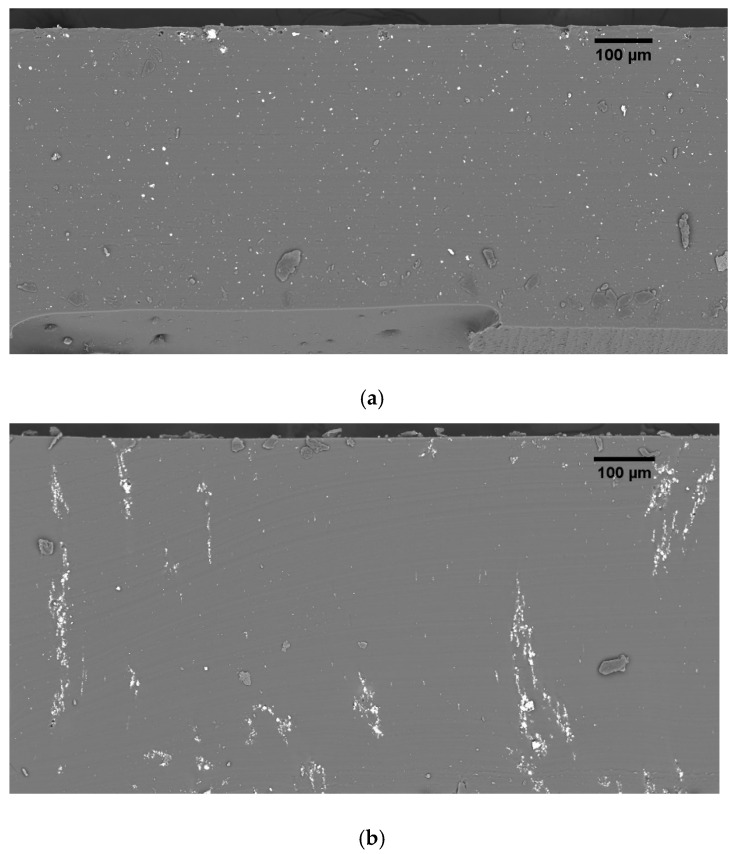
SEM images of the cross section of PZT/PDMS composite with 1% vol of filler: (**a**) 0–3 connectivity; (**b**) 1–3 connectivity.

The particles size was 3.8 µm, with a standard deviation of 1.6. Figure 3 is a picture of the particles taken via SEM in Back scattered electron mode (BSE). SEM pictures of the cross section of 0–3 and 1–3 composite with a volumetric content of 1%vol are illustrated in Figure 4a,b. The analysis highlights the different distribution of the fillers in the two connectivity, being a clear proof of the effect of dielectrophoretic alignment.

### 3.2. Dielectric and Piezoelectric Behavior

**Figure 5:**
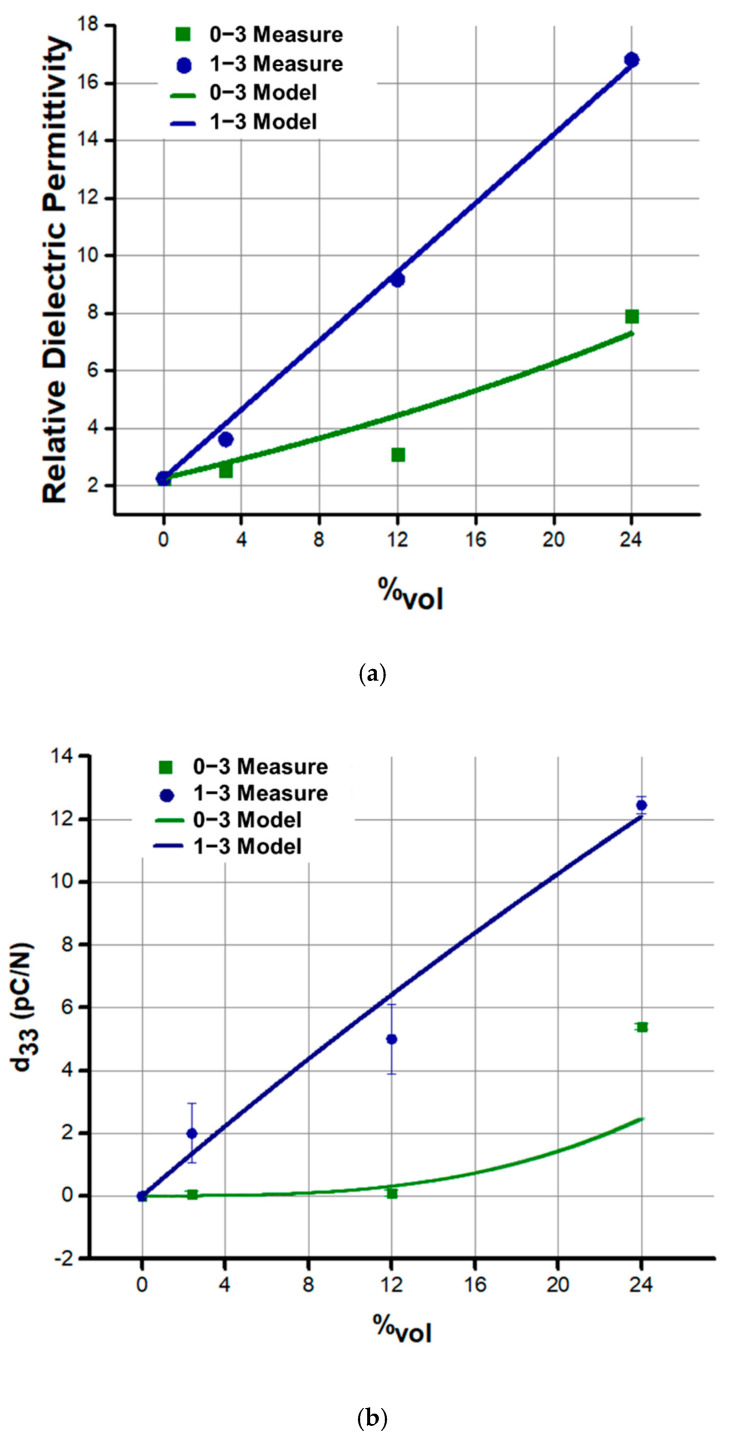

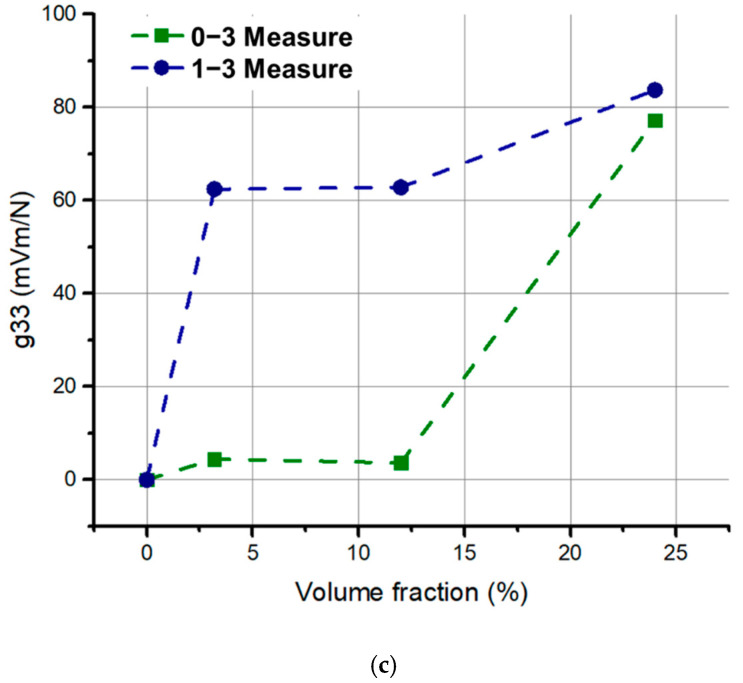
(**a**) Dielectric permittivity, (**b**) piezoelectric charge coefficient, and (**c**) piezoelectric voltage coefficient of 0–3 and 1–3 PZT/PDMS as a function of filler volume content. Comparison between experiment and analytical models was showed in (**a**,**b**).

As expected, the dielectric properties of 1–3 composites were higher at each volume content, as depicted in Figure 5a. The rearrangement of the fillers within the matrix affects the electric field distribution and this leads to an increase of permittivity. The enhancement of permittivity is a proof that dielectrophoresis induces change of composite properties by creating anisotropy. To summarize, it is quite clear that the distribution of the phases has an important effect on the measured dielectric properties. As a result of field-structuring, relative permittivity is increased along the field direction, whatever the volume content. Experimental results were compared to theoretical data established through the models developed by Yamada et al. (for 0–3 connectivity) and Bowen et al. (for 1–3 connectivity) [8,42,46]. Yamada’s model describes 0–3 composites as made by ellipsoidal particles homogeneously dispersed in the matrix and the composite is considered to be mechanical and electrical loss free (Equation (1)). Bowen’s model describes the system as made of perfect cubic particles disposed in aligned chains (Equation (2)). According to the model, the cubic fillers are capacitors in series with the matrix within the chains. These chains are connected in parallel to the matrix region outside of the chains, which is depleted of particles. As shown in Figure 5a, the results follow the trend presumed by the models in both configurations.

The following equation describes Yamada’s model for dielectric properties of 0–3 connectivity composites:

(1) $$\varepsilon_{0–3}=\varepsilon_{p}\left(1+\frac{n\varphi \left(\varepsilon_{f}-\varepsilon_{p}\right)}{{n\varepsilon }_{p}+\left(\varepsilon_{f}-\varepsilon_{p}\right)\left(1-\varphi \right)}\right)$$

where $\varepsilon_{p}$ and $\varepsilon_{f}$ are the real part of the permittivity of the polymer and of the filler respectively; φ is the filler volume content, and n is the inverse of the depolarization factor dependent on the geometry of the particles.

Bowen’s model is given by:

(2) $$\varepsilon_{1–3}=\varphi \left(\frac{{r\;\varepsilon }_{f}{\;\varepsilon }_{p}}{\varepsilon_{f}+\varepsilon_{p}\;r}\right)+\left(1-\varphi \right)\varepsilon_{p\;}$$

where r indicates the ration between the dimension of each filler and the interparticle distance within a chain.

Figure 5b depicts the piezoelectric charge coefficient (d_33_) at different filler volume fractions. As expected, the structured samples exhibit higher d_33_, regardless of the volume fraction content. The 1–3 connectivity induces anisotropy leading to enhanced piezoelectric properties along the column direction. Ceramic particles concentration is enhanced along the thickness direction, while the other directions are depleted of particles [33,42]. Moreover, the distribution of the phases determines the local field intensity, and therefore provokes a certain polarization. In structured composites, for instance, the distribution of the electric field reveals to be more favorable and as a result it allows greater polarization [25,43]. In a composite, the electric field usually concentrate in the polymer matrix rather than in the filler, due to the lower dielectric properties of the polymer. However, in 1–3 configuration, the electric field focuses mostly on the polymer gaps between the particles, making polarization of ferroelectric particles significantly enhanced. In 0–3 connectivity, using the same electric field as in the case of 1–3 counterpart is not sufficient enough to reach the most optimized polarization [25,43].

In this work, the experimental results were compared with the analytical models: Yamada’s model for d_33_ in 0–3 connectivity (Equation (3)), and Van den Ende’s model for d_33_ in 1–3 connectivity (Equation (4)) [8,33].

(3) $$d_{33\;0–3}=\varphi \alpha \frac{{n\varepsilon }_{0-3}}{{n\varepsilon }_{0-3}+\left(\varepsilon_{f}-\varepsilon_{0-3}\right)}d_{33\;f}$$

(4) $$d_{33\;1–3}=\frac{{\left(1+r\right)}^{2}{*\varepsilon }_{p}{*\varphi *Y}_{33\;f}^{\;}{*d}_{33\;f}^{\;}}{(\varepsilon_{f}+{r\varepsilon }_{p})*\left[\left(1+r\varphi \right){*Y}_{33\;f}^{\;}+\left(1-\varphi \right){*\mathrm{rY}}_{p}\right]}$$

where α is the poling ration of the filler,${\;d}_{33\;f}^{\;}$ the piezoelectric charge coefficient of the filler, $\;\;Y_{33\;f}^{\;}$ the elastic modulus of the ceramic inclusions, and ${\;Y}_{p}$ the elastic modulus of the polymer matrix.

As illustrated Figure 5b, empirical data effectively suit the theoretical trends reflecting high reliability of the proposed approach [8,33]. The piezoelectric voltage coefficient, g_33_, was then computed for all the prepared samples, according to Equation (5). This coefficient, indicating the electric field generated by a *piezoelectric material* per unit of mechanical stress, is important to assess a material’s suitability for sensing and energy harvesting applications. All the structured samples lead to higher g_33_ with respect to the non-structured ones (Figure 5c).

(5) $$g_{33}=\frac{d_{33}}{\varepsilon_{0}\varepsilon_{r}}$$

### 3.3. Thermal Study of Piezoelectric Activity and XRD Analysis

**Figure 6:**
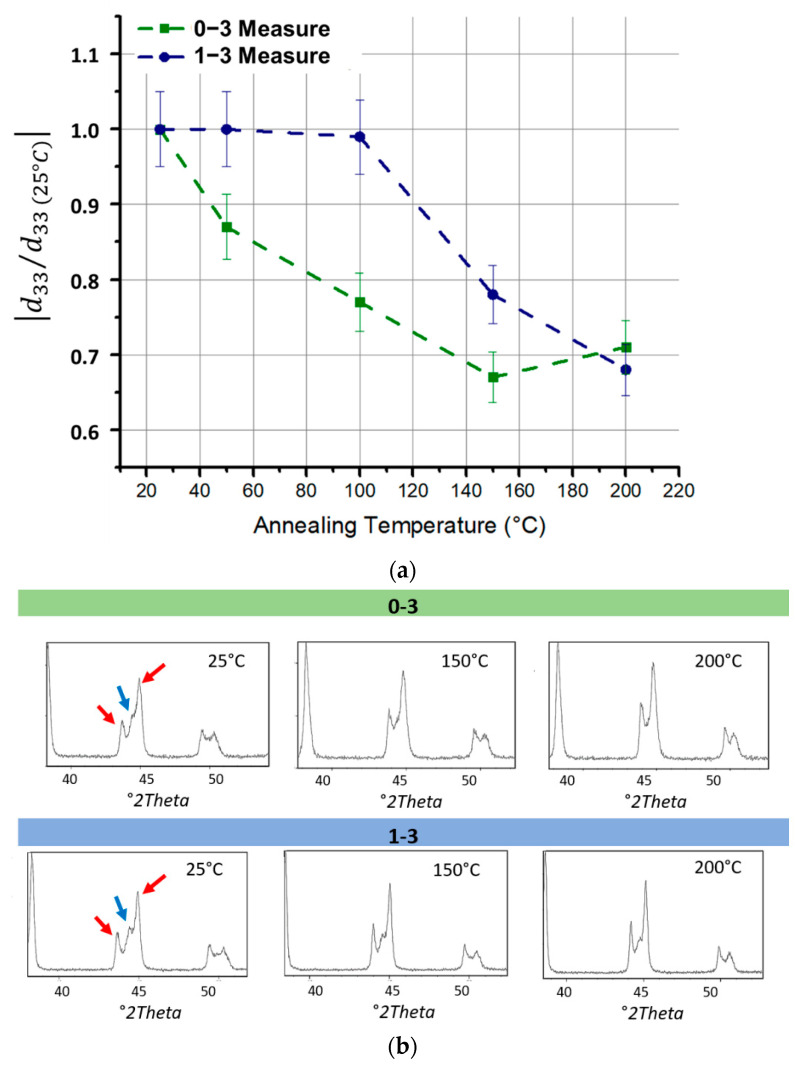
(**a**) Piezoelectric charge coefficient variation with annealing temperature of 0–3 and 1–3 PZT/PDMS 24%vol. The value is normalized with respect to the value at 25 °C. (**b**) X-ray diffraction of 0–3 and 1–3 PZT/PDMS 24%vol at 25 °C, 150 °C and 200 °C. The red arrows indicate the tetragonal ferroelectric phase, while the blue one the rhombohedral phase.

Figure 6a shows the variation with temperature of d_33_ coefficient for 0–3 and 1–3 composites at 24% vol, normalized with respect to its value at 25 °C. In 0–3 composites, the piezoelectric coefficient starts to decrease at 50 °C, while in 1–3 composites it remains constant up to 100 °C, then gradually decreases. Interestingly, a small increase was observed for the 0–3 sample after 150 °C, which is possibly due to the measure error related to the instrument accuracy of around 5%. Figure 6b illustrates the data resulting from the XRD analysis performed at different temperatures (25 °C, 150 °C, and 200 °C). This study allowed to associate the results of the thermal analysis with the crystalline structure of the ceramic filler. At room temperature, PZT has a combination of two crystalline structures, tetragonal and rhombohedral, both ferroelectrics, and therefore with an asymmetry related to piezoelectric behavior, following alignment of the dipoles via poling procedure. The combination of these phases is called the morphotropic phase boundary (MPB). As shown in Figure 6b, the red arrows indicate the peaks of the tetragonal phase, while the blue arrow indicates the peak of the rhombohedral phase. This last peak decreases with increasing temperature and only tetragonal peaks remain at 200 °C. In 0–3 connectivity, the rhombohedral phase peak disappears at lower temperature (i.e., 150 °C) with respect to the 1–3 connectivity. This could justify the earlier decrease of piezoelectric properties. It is worth noting that there is no passage to cubic phase (i.e., corresponding to paraelectric behavior), which in fact only occurs above the Curie temperature (T_c_ ≈ 350 °C). In conclusion, the disappearance of the rhombohedral peak induces a slight decrease (30% approximately) in the piezoelectric properties of the PZT. Nevertheless, the composite keeps a good piezoelectric response up to 200 °C, which reveals to be suitable in a large application of sensing devices.

### 3.4. In-Situ Microscopic Observation

**Figure 7:**
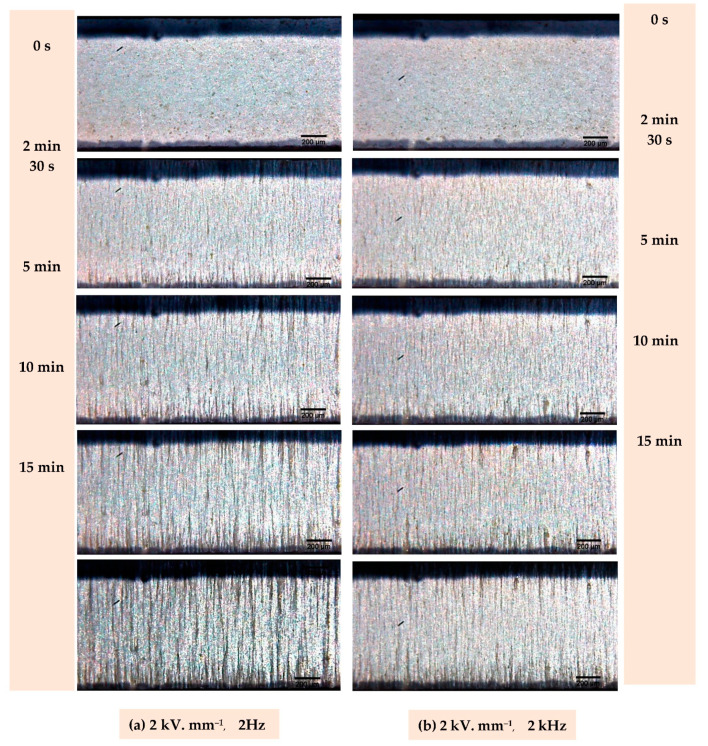
Frame of in-situ microscopic observation during dielectrophoresis performed at 2 kV. mm^–1^ from 0 to 15 min, with frequency of (**a**) 2 Hz, and (**b**) 2 kHz.

Figure 7a,b respectively display the frame of the 1% vol blend structuration subjected to a 2 V µm^–1^ electric field of 2 Hz and 2 kHz, at different moments of the dielectrophoretic process 2 Hz, forming coarser particle’s chain-like structure, reveals to be more effective than 2 kHz, especially after 15 min of process. Different studies clearly indicated that dielectrophoresis is more effective under low frequency (i.e., around 1 to 10 Hz). This behavior is probably due to slow polarization of the ceramic particles, which makes them unable to follow the electric field at a high dynamic (e.g., 100 Hz to 1 kHz). However, frequencies lower than 1 Hz were not considered as they would lead to inefficient structuration [25]. Indeed, at extremely low frequencies, the ionic species have time to redistribute themselves into charge layers near the electrodes, provoking the voltage drop and, therefore, inhibiting chain formation. Regarding the electric field amplitude, according to the literature, an excessively high input level would generate turbulence and consequently destroy the columns. Moreover, the value of electric field amplitude is limited by the dielectric breakdown of the polymer [25]. On the other hand, this value should be high enough to successfully align the particles in chain-like structure. Accordingly, 2 V µm^−1^ seems to be the most suitable choice to achieve this compromise.

In order to validate this qualitative observation, a sample made with 24%vol with 1–3 connectivity was produced using an electric field of 2 V µm^−1^ and 2 Hz of frequency. The d_33_ coefficient of the 1–3 sample (after being poled) was measured and compared to the same volume content sample but produced at 2 kHz, in order to confirm the best tuning parameter used in dielectrophoresis. The result reveals that the d_33_ value of the sample carried out at 2 Hz reaches 16 pC N^−1^, corresponding to an improvement of around 30% with respect to the 2 kHz counterpart. This observation is coherent to the microscopic analysis of Figure 4.

### 3.5. Overview of the Piezoelectric Properties: Potential Application in Stretchable Electronics

**Figure 8:**
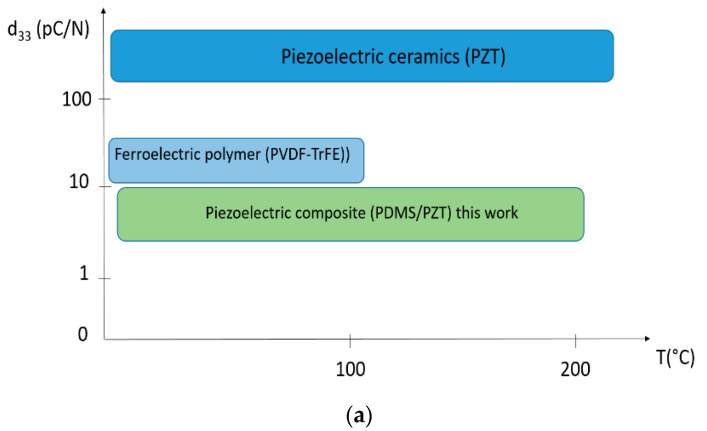

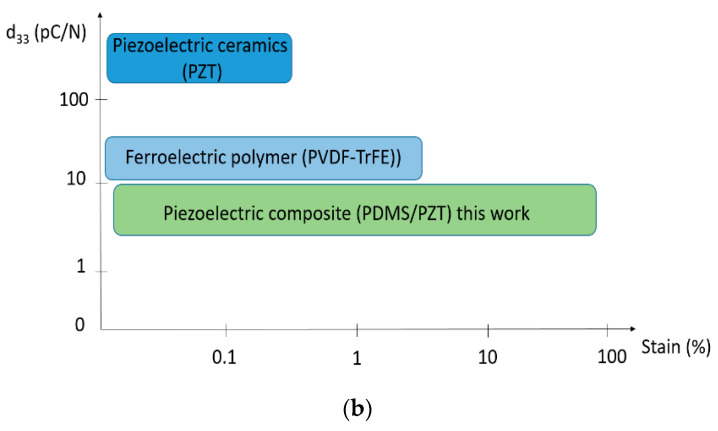
Overview of three typical piezoelectric materials (ceramic, polymer, composite) with their piezoelectric charge coefficient: (**a**) versus temperature, and (**b**) versus mechanical strain.

**Figure 9:**
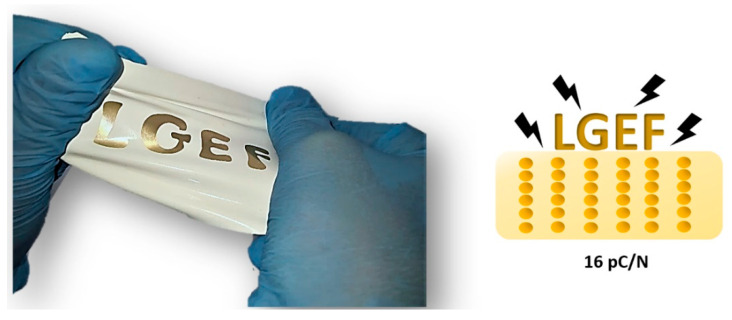
Picture of stretched piezoelectric sensor developed in this study.

The proposed inorganic/organic piezoelectric composite has a great potential in the field of stretchable electronics and elastronic [47,48]. Figure 8a,b compares the piezoelectric sensitivity between our composite and other classical piezoelectric materials in terms of working temperature and mechanical strain, respectively. It is believed that our composite can undergo high strain and large temperature with respect to piezoelectric ceramics and ferroelectric polymer. Such properties could be required for many applications in different fields, for instance aerospace as elastomeric sensors for monitoring components [49,50]. As a matter of fact, sensing systems are usually made by hard materials and all their functionalities rely only on the electronic architecture and on the software embedded in the device chip. This approach has serious drawbacks since a rigid structure imposes severe limitations on the device application domains (mechanical deformation less than 1 or 2%) [51]. In some cases, it is possible to overcome these limitations by augmenting the complexity either of the device physical structure or of the embedded software. This, however, results in an unwanted great increase in the final cost or in degraded performances. Thus, it is strategic to develop materials that facilitate disruptive or transformative changes while being fully compatible with standard electronics [52]. Hence, our proposed material does not act just as static structural component, but actively interacts with the environment. In other words, it can respond to external stimuli altering its physical properties in a way that can be exploited to improve the functionalities of a device. Figure 9 depicts a photograph of the stretched sample associated with its typical piezoelectric response.

From a physical point of view, the proposed approach involves enhancement of the filler concentration along the alignment direction. In practice, all particles disposed in columns along the electric field lines, giving raise to enhanced electric polarizability of the dielectric material. It has been revealed that the 1–3 connectivity polarizes more in response to an applied electric field than the corresponding 0–3, which explains why the permittivity is higher in the case of structured 1–3 sample. In practice, lower electric field is possible to fully polarize the aligned fillers within the matrix, while is not in a 0–3 composite. Actually, the matrix of this configuration substantially attract the electric field, inducing extremely poor amount of electric field in the filler [53,54]. Nonetheless, in 1–3 configuration, the enhancement of the electric field in the matrix gaps between the particles is high enough to reach full polarization of the particles. Consequently, under the same poling condition, the piezoelectric activity of the 1–3 composite significantly improved as opposed to the 0–3 counterpart.

## 4. Conclusions

In this work, we developed piezoelectric composites made of PZT microparticles, embedded in Silicone PDMS matrix, with two different internal connectivity, 0–3 and 1–3. SEM analysis provided a clear image of dielectrophoretic effect on the blend, confirming that such a process can successfully drive particles in column-like structure. Dielectric and the piezoelectric characterization of 0–3 and 1–3 composites pointed out that the 1–3 connectivity leads to a 2-fold improvement in the dielectric permittivity (ε_r_), and more than 2.5-fold in the piezoelectric charge coefficient (d_33_), as opposed to the 0–3 counterpart for 24% vol of filler concentration. Whatever the material connectivity (either 1–3 or 0–3), experimental trends of ε_r_ and d_33_ were in good agreement with theoretical models. To access the behavior of composites at high temperature, a study based on thermal stability coupled with XRD analysis were performed, allowing to verify the existence of ferroelectric crystalline structure until 200 °C. Finally, an in-situ microscopy analysis led to the best parameters for alignment procedure. It was pointed out that a low frequency of 2Hz gave raise to improved d_33_ piezoelectric coefficient of 30% with respect to 2 kHz, which was conform with the microscopic observation.

In conclusion, the developed composite, whose piezoelectric sensitivity (16 pC N^−1^) is close to the one of conventional ferroelectric polymers (20 pC N^−1^), was successfully produced via dielectrophoresis. Besides its high piezoelectric activity, further advantages that make this material very attractive in several fields (structural health monitoring, energy harvesting, etc.) are cost effectiveness, easy processability, low poling electric field requirement, integrability in complex structures thanks to its flexibility, and ability to survive high temperature environments.

## Data Availability

Not applicable.
